# A case of bovine raw milk contamination with *Listeria monocytogenes*

**DOI:** 10.1186/2046-0481-65-13

**Published:** 2012-07-06

**Authors:** Karen Hunt, Niall Drummond, Mary Murphy, Francis Butler, Jim Buckley, Kieran Jordan

**Affiliations:** 1 Teagasc Food Research Centre, Moorepark, Fermoy, Co. Cork, Ireland; 2 Cork County Council Veterinary Food Safety Laboratories, Iniscarra, Co. Cork, Ireland; 3 Veterinary Sciences Centre, School of Agriculture, Food Science and Veterinary Medicine, University College Dublin, Belfield, Dublin

**Keywords:** *Listeria monocytogenes*, Raw milk, Subclinical mastitis, Milk quality

## Abstract

During routine sampling of bulk raw milk on a dairy farm, the pathogenic bacteria *Listeria monocytogenes* was found to be a contaminant, at numbers < 100 cfu/ml. A strain with an indistinguishable pulsed-field gel electrophoresis pattern was isolated from the bulk milk two months later. Environmental swabs taken at the dairy environment were negative for the presence of *L. monocytogenes*, indicating a possible case of excretion of the *L. monocytogenes* directly into the milk. Milk samples were collected from the individual cows and analysed, resulting in the identification of *L. monocytogenes* excretion (at 280 cfu/ml) from one of the 4 mammary quarters of one dairy cow out of 180. When the infected cow was isolated from the herd, no *L. monocytogenes* was detected from the remaining herd. The pulsed-field gel electrophoresis pattern of the strain from the individual cow was indistinguishable from that originally isolated from the bulk milk. The infected cow did not show any clinical signs of disease, nor did the appearance of the milk have any physical abnormalities. Antibiotic treatment of the infected mammary quarter was found to be ineffective. This study shows that there can be risks associated with direct contamination of raw milk with *L. monocytogenes*.

## Background

*Listeria monocytogenes* is a pathogenic bacterium that can cause Listeriosis in humans and various animal species. In humans, foodborne *L. monocytogenes* causes large outbreaks of Listeriosis, with a mortality rate of 9% to 44% [1]. In a wide variety of host animals *Listeria* infection has been confirmed in more than 40 species of domestic and wild animals including birds. The most susceptible domestic species are sheep, goats and cattle. Listeriosis manifests itself clinically in ruminants as encephalitis, neo-natal mortality (abortion) and septicaemia. The most common clinical form in cattle is encephalitis, in general, small numbers being affected (8-10% of the herd) with the animals surviving from 4–14 days. In animals, susceptibility to infection with *L. monocytogenes* has been attributed to decreased cell-mediated immunity associated with advanced pregnancy [2]. *L. monocytogenes* has the ability to invade both phagocytic and non-phagocytic cells, to survive and replicate intra cellularly, and to transfer from cell to cell without exposure to humeral defence mechanisms.

In raw milk and the dairy environment, the source of *L. monocytogenes* contamination is mainly from poor silage and bedding [3,4]. On the farm, contamination of *L. monocytogenes* can spread from the environment to the animals and also from animal to animal [2,5-7]. Contamination of milking equipment with bovine faeces can also occur [8]. During storage of raw milk on the farm, *L. monocytogenes* can grow and multiply, even at refrigerated conditions [9].

Udder infection with *L. monocytogenes* is most commonly reported in sheep and goats [10]; *L. monocytogenes* bovine mastitis is less commonly reported where sub-clinical mastitis in cows can go undetected [11-16], where their milk remains visually unchanged, and with no clinical signs contamination can normally persist even after treatment [14]. Most cases of human listeriosis are foodborne related [17]. The occurrence of *L. monocytogenes* in raw milk was reported as 4.8%, 6%, 3.4% and 6%, respectively [6,18-20]. Raw milk can be contaminated from the environment or by direct excretion into the milk, therefore, consumption of raw milk is associated with increased risk factors. None of the above studies indicated the source of milk contamination, but it was most likely from the environment.

The aim of this study was to identify the source of *L. monocytogenes* that was contaminating bulk milk at farm level.

## Materials and methods

### Environmental swabbing of the dairy environment

Environmental samples were collected from non-milk contact sampling sites including pipes, tanks, drains, floors, and walls. Environmental samples from milk contact points were collected from bulk milk filters and from a milk spill area on the floor of the dairy beside the bulk tank. All swab samples and milk filters were collected as previously described [6].

### Milk sampling

To investigate *L. monocytogenes* contamination of milk, the herd of 180 individual cows was divided into 9 groups with 20 cows in each group. A composite sample was taken aseptically, in sterile containers, from each group and analysed. Within each positive sample from the groups, each of the 20 individual cow’s milk was sampled and analysed. Where milk from a cow was positive, milk from the four quarters of the mammary gland was sampled and analysed.

### *L. monocytogenes* analysis

The presence/absence of *L. monocytogenes* in all milk and environmental samples was analysed using an AFNOR validated “One step enrichment broth” method (Oxoid FT0401). This AFNOR validated method has been shown to give equivalent results to ISO (11290–1:1997). Milk samples were enumerated for *L. monocytogenes* by direct plating of the milk onto selective agar (Brilliance™ Listeria Agar; Oxoid CM1080). Typical *L. monocytogenes* colonies (which are blue-green with a surrounding precipitate) were isolated and purified by re-streaking on ALOA agar (Agosti & Ottaviani *Listeria* Agar; LabM, Lancashire, UK, HAL010), followed by streaking on tryptone soy agar (TSA). Single pure isolated colonies were grown overnight in tryptone soy broth (TSB) and frozen in cryovials in a glycerol/TSB mixture at −20°C.

### Milk quality analysis

Bulk milk and *L. monocytogenes* contaminated milk samples were tested for quality parameters. The pH was measured using BS770:5:1975 with an Orion pH meter model 420A. Enumeration of total bacterial counts using ISO 6610:1992 method with Milk Plate Count Agar (Oxoid CM0681), coliforms using method ISO 4831:2006 on VRBL agar (Oxoid CM0968) and *S. aureus* using method ISO 6888–2 with Baird Parker Rabbit Plasma Fibrinogen agar (Oxoid CM0961). The fat, protein, lactose, casein and Somatic Cell Count (SCC) were measured by infrared analysis (Milkoscan 605, Foss Electric, Denmark).

### Molecular characterisation of *L. monocytogenes* isolates

All purified isolates were confirmed as *L. monocytogenes* using Real-Time polymerase chain reaction (RT-PCR) [21,22]. Pulsed-Field Gel Electrophoresis (PFGE) of all *L. monocytogenes* isolates was performed with the restriction enzymes *Asc*I and *Apa*I, in two separate experiments, using the standard PulseNet protocol [23]. Serotyping of the isolates was performed using a combination of antibodies and serotype-specific PCR [24].

### Examination of cows

The infected cow under examination was physically examined and medically treated by a veterinary surgeon. The treatment included intra-mammary injection of 4 tubes of Sinulux, at 12 hour intervals. This was followed by a five day treatment with Tylosil and a subsequent five day treatment with Oxytetracycline. After antibiotic treatment the infected animal’s milk continued to test positive for *L. monocytogenes.*

## Results

### Environment sampling

All environmental swabs were negative for *L. monocytogenes,* except the milk filters from the bulk tank and the floor of the dairy beside the bulk tank. The isolates obtained had indistinguishable PFGE profiles from the original milk isolate.

### *L. monocytogenes* microbiological analysis

Once the cow that was excreting *L. monocytogenes* into the milk was identified, the milk from individual quarters was examined (Table 1). *L. monocytogenes* was excreted from only one quarter, and this quarter also had a higher Somatic Cell Count (SCC) than the other quarters, although it was still within acceptable limits. Detailed analysis of the milk was undertaken on several days (Table 2). The milk was positive for *L. monocytogenes* on all dates tested. In addition, TBC and SCC were elevated. Fat, protein and lactose concentrations were generally within accepted limits.

**Table 1:** Milk analysis results from the four quarters of the mammary gland, from the Listeria infected cow

Sample	*Listeria*Enrichment	*Listeria*cfu/ml	SCCx 10^3^/ml
Front right	pos	280	207
Front left	neg	<10	79
Back left	neg	<10	9
Back right	neg	<10	4

**Table 2:** Milk analysis results from the Listeria infected milk and the bulk milk (without the infected cow)

Date	Milk source	ListeriaEnrichment	Listeriacfu/ml	Total counts(cfu/ml)	SCCX 10^3^/ml	Fat(%)	Protein(%)	Lactose(%)
18/07/11	Infected cow	pos	<10	ND	387	3.19	3.11	4.71
26/07/11	Infected cow	pos	20	98000	459	2.81	2.98	4.60
28/07/11	Infected cow	pos	307	16000	469	3.26	2.97	4.66
02/08/11	Infected cow	pos	50	77000	427	3.52	2.97	4.65
03/08/11	Infected cow	pos	520	140000	727	4.10	2.81	4.49
03/08/11	Infected cow	pos	520	140000	727	4.10	2.81	4.49
22/07/11	Bulk milk	neg	neg	ND	125	3.71	3.27	4.80
16/08/11	Bulk milk	neg	neg	ND	183	4.13	3.31	4.67

ND not determined.

### Milk quality

The average pH of infected bulk milk was pH 6.81(n = 5). *L. monocytogenes* contaminated milk samples tested had enumeration results of <10 cfu/ml for *coliforms* and *S aureus*. Table 2 shows total bacteria count values for *L. monocytogenes* infected milk and the fat, protein lactose, casein and SSC results for both the infected milk and the non infected bulk milk.

### *L. monocytogenes* characterisation

**Figure 1:**
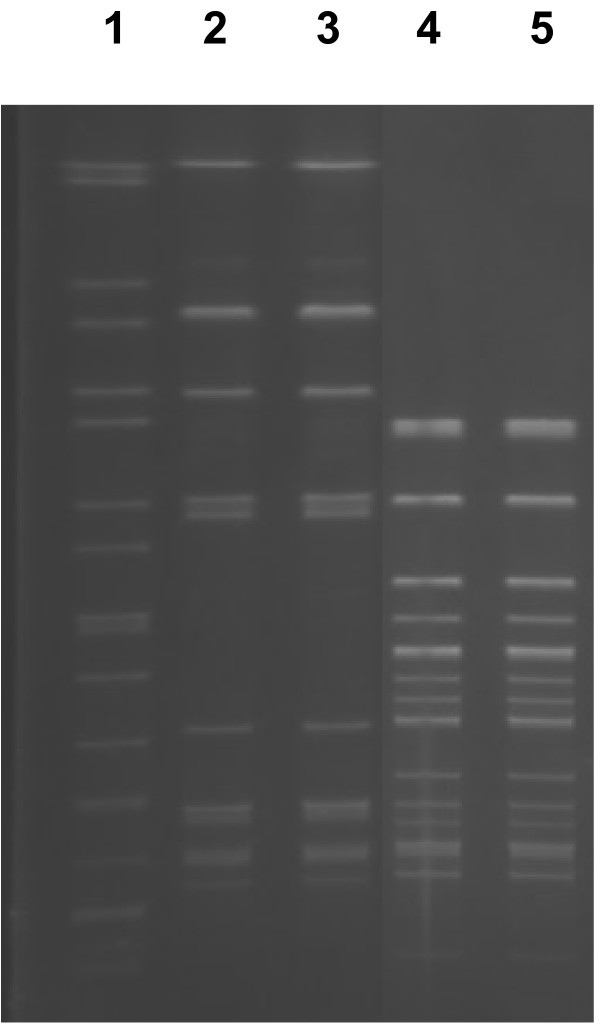
**PFGE profiles of *L. monocytogenes *from bulk tank milk.**

All *L. monocytogenes* strains isolated in this study had indistinguishable PFGE profiles (Figure 1) and were all serotype 1/2a.

## Discussion

*L. monocytogenes* has been described as a “*less common cause”* of bovine environmental mastitis and may often be associated with the accidental introduction of the organism during intramammary infusion [25]. Mild persistent, antibiotic resistant, low grade infections have been documented in cattle and may result in decreased production from infected mammary tissue over time. In addition, *L. monocytogenes* infection and secretion may have potential public health implications for susceptible consumers [2], if raw milk is consumed. *L. monocytogenes* has been isolated from different species and from soil, plants, mud, pasture, waste water and streams. Cattle and many other animal species excrete *L. monocytogenes* in their faeces. Controls to reduce the level of contamination and infection include such measures as the pasteurisation of milk, rodent control and common practices of dairy husbandry, personal hygiene and environmental management. Animals with encephalitis or those that have aborted should be isolated and all in contact material including placenta and foetuses aseptically removed and destroyed [13].

In cases of *L. monocytogenes* contamination of milk, the most likely source of the *listeria* is from the environment post-milking. Direct excretion of *L. monocytogenes* into the milk, i.e., clinical or sub-clinical mastitis is rare.

In this case*, L. monocytogenes* contamination of a bulk raw milk sample led to an investigation of the source of contamination. Environmental swabs from the dairy environment were negative, except for milk contact surfaces. It is possible that the contamination from the dairy floor led to contamination of the milk, but with milk contamination lasting for several months, this is unlikely. Previous studies of *L. monocytogenes* contamination of raw milk found that animal faeces, poor quality of feed and general lack of hygiene on the dairy farm are factors associated with contamination [3,26,27]. Biofilms formation has been known to cause adhesion and persistence of *L. monocytogenes* onto equipment [28]. Levels of hygiene on the farm involved in this study were visually very good, and all non-milk contact dairy environmental swabs were negative, indicating the source of contamination was direct excretion into the milk. Following a visual inspection, none of the cows in the herd had any physical signs of infection.

The *L. monocytogenes* contamination in the bulk milk was further investigated in herd milk testing. Using segregated composite bulk milk sampling (20 cows per sample), one mammary quarter from one cow infected, without any clinical or sub-clinical signs of mastitis was eventually identified [29]. This infected cow was segregated from the herd; the bulk milk was then *L. monocytogenes* negative. After the cow was segregated from the herd, the SCC results of the contaminated milk samples increased (Table 2), indicating sub clinical mastitis, although the stress of separation could also result in increased SCC counts. In dairy cattle herds with mastitis it is found that in almost all cases where Listeria occurred, only one quarter is infected, and 95% of cases yielded a pure culture [30], similar to the results of this study.

The quality of the infected milk was variable with regards to TBC, fat, protein and lactose levels (Table 2). When the SCC was measured initially, the values were elevated slightly in the contaminated quarter, but when diluted with the 3 non-contaminated quarters the average ~50,000 cells/ml, is well within expected limits for good quality milk. Even the contaminated quarter was acceptable at 207,000 cells/ml. When the cow was isolated from the herd and in a new environment, this could have imposed an additional stress where the SCC was raised to sub-clinical levels of >400,000 cells/ml (current EU limit as Annex III, Section IX, Chapter I,

Part III, to Regulation (EC) No 853/2004.) However, no visual defects in the milk, nor clinical signs of infection were seen in the infected cow during this study.

Testing of the infected cow and the remaining bulk milk was carried out for a further 2 months (6 months from initial identification of the problem). Over the 6 months of testing, all the strains of *L. monocytogenes* from the infected milk were indistinguishable when characterised by PFGE (Figure 1) and serotyping, indicating that the same strain of *L. monocytogenes* was persistently present in the infected milk. The persistence of infection was also reported by Bourry 1995 [16] with sub-clinical mastitis. With sub-clinical listeria mastitis, cows not showing any clinical signs go undetected where they may produce milk with normal appearance containing large numbers of pathogenic *L. monocytogenes*[15,16]. Clinical mastitis is, by definition, abnormal milk and no reference to SCC is required [31]. The presence of flakes, clots, or other gross alterations in appearance of quarter milk is evidence of clinical mastitis and is by definition, abnormal milk.

## Conclusions

In this study we find bulk raw milk to be directly contaminated from a case of *L. monocytogenes* infection in a dairy cow. The SCC was slightly elevated from the infective quarter with no visual defects in the milk, or clinical signs seen of infection in the cow. This *L. monocytogenes* strain was isolated from all milk samples and by molecular characterisation, found to be a pure and persistent isolate over a 6 month period, even after antibiotic treatment.

## Competing interests

The authors declare that they have no competing interests.

## Author’ contributions

KH, KJ and ND carried out sampling and molecular genetic studies. KJ, MM, JB and FB planned the work and drafted the manuscript. All authors read and approved the final manuscript.
